# A rehabilitation intervention to improve recovery after delirium in older people: mixed methods process evaluation of the RecoverED multi-site feasibility study

**DOI:** 10.1186/s12877-026-07314-z

**Published:** 2026-03-31

**Authors:** Shruti Raghuraman, Aseel Mahmoud, Alison Bingham, Abigail Laverick, Kirstie Chandler, Abby O’Connell, Jinpil Um, Obioha C Ukoumunne, Elizabeth Goodwin, Annie Hawton, Lesley Collier, Sarah Joanna Richardson, Alasdair Maclullich, Rachael Litherland, Victoria A Goodwin, Linda Clare, Louise Allan, Sarah Morgan-Trimmer

**Affiliations:** 1 Health and Community Sciences, University of Exeter, Exeter, UK; 2 Exeter Clinical Trials Unit, University of Exeter, Exeter, UK; 3 NIHR Applied Research Collaboration South West Peninsula, University of Exeter, Exeter, UK; 4 Health Economics Group, University of Exeter Medical School, University of Exeter, Exeter, UK; 5 Faculty of Health and Well‑Being, University of Winchester, Winchester, UK; 6 AGE Research Group, Newcastle upon Tyne Hospitals NHS Foundation Trust, Cumbria Northumberland Tyne and Wear NHS Foundation Trust, Newcastle University, Newcastle Upon Tyne, UK; 7 Faculty of Medical Sciences, NIHR Newcastle Biomedical Research Centre, Newcastle Upon Tyne, UK; 8 Scottish Hip Fracture Audit (SHFA), NHS National Services Scotland, Edinburgh, UK; 9 Ageing and Health Group, Usher Institute, University of Edinburgh, Edinburgh, UK; 10 Innovations in Dementia, Exeter, UK; 11 School of Primary Care, Population Sciences and Medical Education, University of Southampton, Southampton, UK; 12 Faculty of Health and Life Sciences, University of Exeter, Exeter, UK

**Keywords:** Implementation fidelity, Acceptability, Community rehabilitation, Healthcare delivery, Delirium

## Abstract

**Objective:**

A mixed-methods process evaluation was conducted alongside a multi-site feasibility trial of RecoverED, a multicomponent delirium rehabilitation intervention for older people in post-acute settings. A modified Conceptual Model for Implementation Fidelity was used. Findings on implementation and acceptability are presented.

**Design and methods:**

Older adults with delirium, their carers, and trained healthcare professionals (HCPs) from six UK NHS hospitals participated. Adherence to content, dose, and coverage, alongside moderating factors such as recruitment, context, and delivery quality, were examined. Findings from in-depth interviews, focus groups, trial documentation, and training logs were triangulated.

**Results:**

Nineteen participant-carer pairs were recruited to the study. Post-intervention, five older adults, nine carers, and eight HCPs participated in interviews, while seven HCPs took part in focus groups. Adherence to content was challenging to assess due to the intervention’s personalised nature. Psychosocial support was delivered more frequently than planned. Participant-led goals were highly valued, with strong engagement and perceived benefit. Implementation was largely as intended. Most withdrawals (*N* = 10) were attributed to complex needs. No participants from minority ethnic backgrounds were recruited.

**Conclusions:**

The RecoverED intervention was acceptable, though recruitment and retention challenges necessitate caution when interpreting acceptability and fidelity to dose and coverage. Implementation fidelity was high and well-received.

## Introduction

Delirium is an acute, neuropsychiatric syndrome and is highly prevalent in older people across different care settings [4]. Delirium is associated with a range of poor outcomes including mortality [3, 5, 21, 35, 36, 42], especially when superimposed on dementia [11, 25], or when there is partial or no recovery [8, 9], such as when delirium is persistent, or some features of delirium are present.

The National Institute for Health and Care Excellence (NICE) issued a call for research into evidence-based, multi-component treatment interventions to help recovery from delirium [29]. There is currently no research that addresses the long-term support and recovery needs of older people with current or persistent delirium once they have been discharged from hospital [5, 23].

In response to this research and practice gap, the RecoverED (Recovery after an Episode of Delirium) project sought to develop and evaluate a novel, complex, multicomponent rehabilitation intervention for older people with delirium and their carers. The intervention was tested in a multi-centre, single arm feasibility study with an embedded process evaluation to assess the feasibility, acceptability and implementation fidelity of the intervention [2]. The intervention development process and findings on feasibility are presented elsewhere; this paper focuses on the acceptability and implementation fidelity of the intervention.

Acceptability is ‘the perception among implementation stakeholders that a given treatment, service, practice, or innovation is agreeable, palatable, or satisfactory’ ([33], p. 67). The acceptability of a novel intervention can impact on other implementation considerations such as adoption, penetration and sustainability [33]. Carroll et al. [6] An evaluation of acceptability will facilitate understanding of engagement or adoption of a new intervention. This is pertinent to the evaluation of complex interventions, as greater complexity may pose greater risks to adoption [14].

Implementation fidelity refers to the extent to which interventions are delivered as intended by their developers [7]. Evaluating fidelity helps researchers assess the impact of an intervention on desired outcomes, beyond estimating its effectiveness. This aligns with the updated Medical Research Council (MRC) guidance on developing and evaluating complex interventions, which emphasises the role of process evaluations in assessing fidelity, understanding mechanisms of change, and identifying contextual factors affecting outcomes [10, 30, 37]. Embedded process evaluations in effectiveness trials (e.g., feasibility studies or RCTs) can identify discrepancies between intended and actual delivery, aiding the refinement of programme theories [6]. This is consistent with the MRC’s recommendation to theorize interventions at the start and refine them through evaluation phases [37]. Given the multicomponent nature of complex interventions, evaluating fidelity is crucial, as they are more susceptible to variations in implementation [24]. Process evaluations can also identify strategies to improve fidelity and adapt implementation to local contexts [7].

### Conceptual model of implementation fidelity

Carroll et al. [7] developed a multi-faceted conceptual framework to evaluate implementation fidelity in complex interventions, with detailed descriptions available in supplementary materials. A review of 20 empirical studies across disciplines such as public health, medicine, and education found the framework effective for measuring both acceptability and fidelity, with its identified moderators impacting outcomes [6]. In this study, we used a modified version of Carroll’s framework, adding two moderators, context and recruitment [17]. We also excluded ‘comprehensiveness of policy description’ due to a lack of clear guidance from the original authors on its application, and because it was not directly relevant to the aims of our study.

**Fig. 1:**
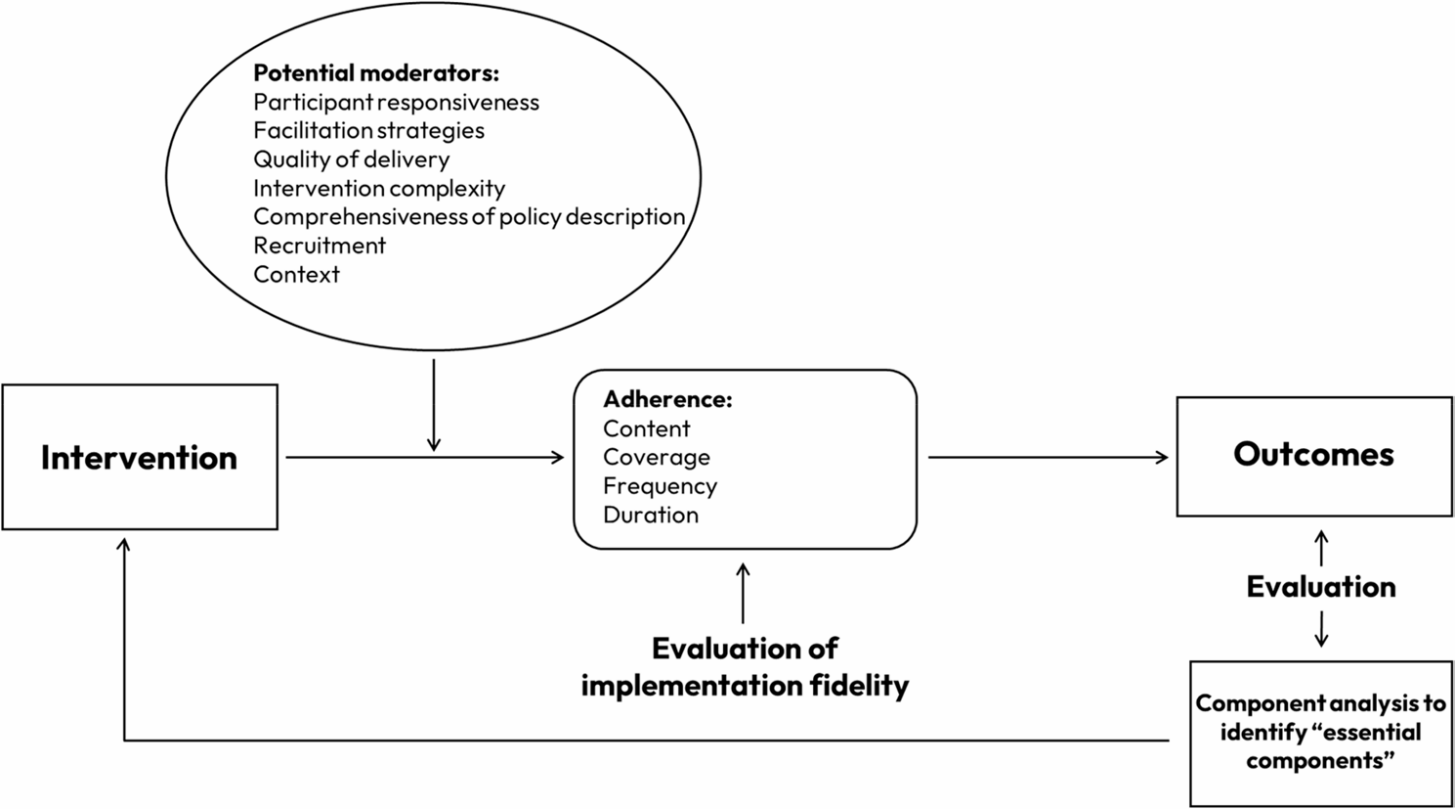
The modified conceptual framework for implementation fidelity, originally from Carroll et al. [7, 17]

The modified framework is presented in Fig. 1.

### Research aims and questions

The aim of this paper is to evaluate the feasibility of implementing the RecoverED intervention with fidelity and to assess its acceptability among recipients and healthcare professionals who deliver it. The main research questions are –

1. Can the RecoverED intervention be delivered as intended, and what factors influence the fidelity of its implementation?
2. Is the RecoverED intervention acceptable to older people with delirium, their carers, and healthcare professionals?

## Methods

### The RecoverED intervention

**Fig. 2:**
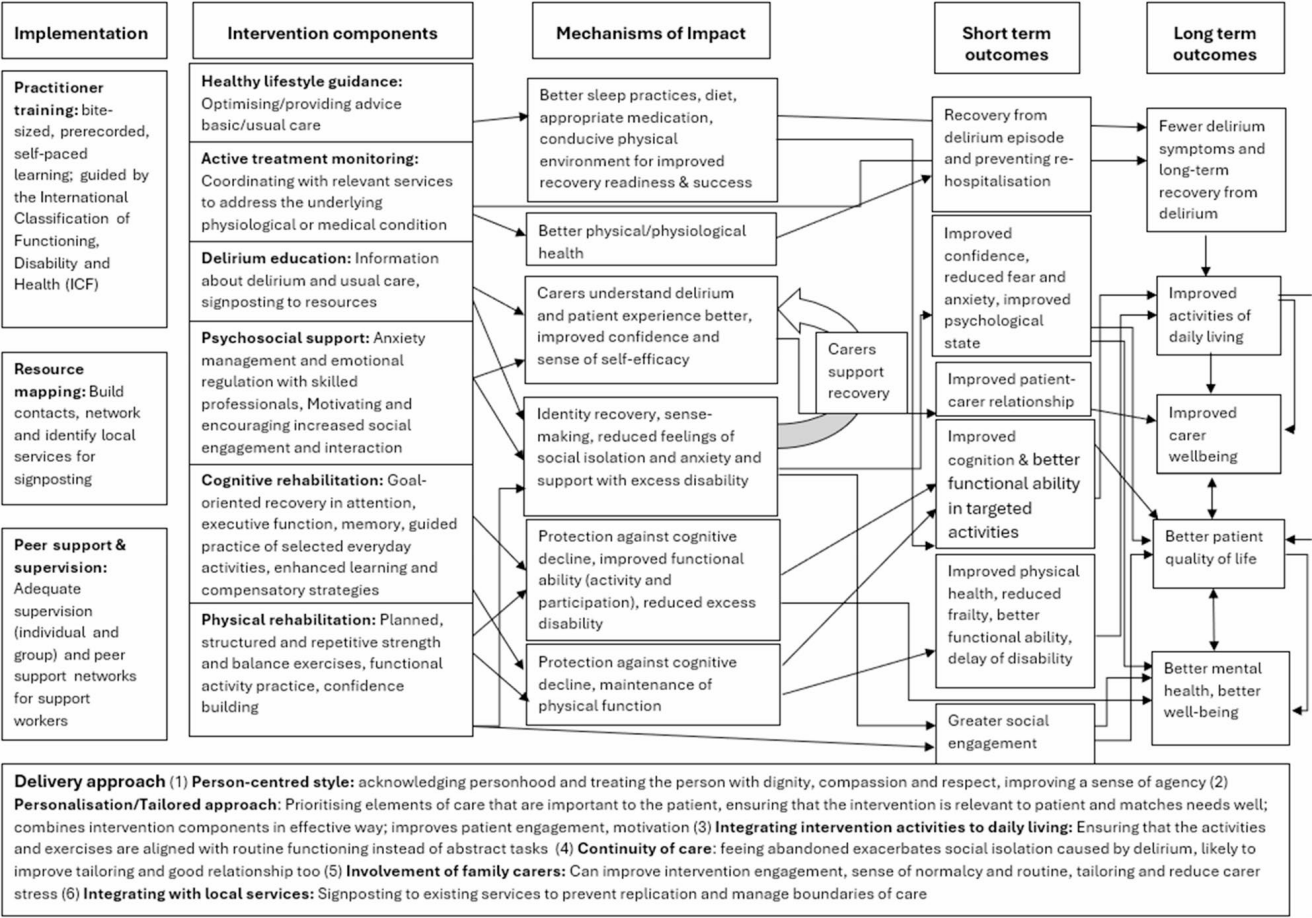
RecoverED logic model

The RecoverED intervention was developed in accordance with the MRC guidance on developing and evaluating complex interventions [37]. A programme theory and logic model were developed using a realist approach [31]. The logic model is presented in Fig. 2.

The programme theory also specified that the intervention must be delivered using a particular style of delivery or approach, which includes –

1. Person-centred style.
2. Tailoring and personalisation.
3. Carer involvement.
4. Relationship continuity.
5. Integration of activities to daily living.
6. Integration with usual care and local services.

The intervention began with an initial assessment conducted by a physiotherapist (PT) or occupational therapist (OT) and a rehabilitation support worker (RSW) within 14 days of discharge. Patient-led goals were established during the assessment, with input from the carer. The intervention was delivered over 10 sessions across 12 weeks, with a midway review to reassess goals, progress, and recovery. Further details on the intervention design, planning, and delivery are provided elsewhere (ref.).

### Design

A multi-centre, single-arm feasibility study was conducted from 2023 to 2024, with details published elsewhere [2]. Nineteen participant pairs (patients and carers) were recruited from six NHS Trusts across the UK. A mixed methods process evaluation assessed implementation fidelity using a modified version of the conceptual model for implementation fidelity [7, 17].

A Patient and Public Involvement and Engagement (PPIE) group collaborated with the study teams to offer valuable input on the process evaluation design, data collection, and analysis.

### Study sites

Older people with delirium were recruited between June 2023 and March 2024 from six NHS Trusts in England and Scotland, selected for their demographic and geographic diversity (Devon, Birmingham, London, Nottingham, Edinburgh, and Newcastle). Across the six sites, 419 patients were screened for delirium, with 36 meeting eligibility criteria (9%; 95% CI: 6%–12%). Of these, 19 patient–carer dyads provided consent to take part (53%; 95% CI: 35%–70%). Thirteen participants went on to initiate the intervention (68%; 95% CI: 43%–87%), and 10 completed the final follow-up assessment (53%; 95% CI: 29%–76%).The intervention was delivered by a trained community rehabilitation team who received comprehensive training and a manual outlining intervention planning and delivery.

### Trial participants

Patient-participants (hereafter referred to as ‘participants’) were eligible for the trial if they: (i) were aged over 65 years, (ii) had a clinical diagnosis of delirium while admitted to a hospital, (iii) had a carer (a family member or friend) and (iv) would be living in their home (not in residential/nursing care) after discharge. Patient-participants and carer-participants (a family member or friend) who were enrolled in the study were simultaneously recruited for the process evaluation, and they could either consent or decline participation in the process evaluation while remaining in the main intervention trial. The study aimed to recruit approximately 60 participant–carer pairs, as specified in the published protocol, to allow estimation of key feasibility parameters with reasonable precision [2].

### Professional participants

Health and social care professionals (HCPs) who were involved in the trial (RSWs, OTs, PTs, service managers and clinical leads) were contacted via email and received a written participant information sheet before providing voluntary informed consent to participate in the process evaluation.

### Data sources and collection

Data were collected from multiple stakeholders between June 2023 and July 2024 to capture diverse perspectives, providing a nuanced view of fidelity and acceptability [13]. A mixed-methods triangulation design was used to consolidate findings and provide a comprehensive account of implementation fidelity. The use of multiple data collection methods is recognised as a strength in previous fidelity assessment studies [18]. Table 1 presents a detailed description of the data sources and data type.

**Table 1:** Description of data sources

Data source	Data source description	Type of data
Case Report Forms (CRFs):	- CRFs were completed by the study team at each site, capturing consent, intervention sessions, planned and delivered activities, withdrawals, and participants’ sociodemographic data. - Data were entered into a secure online database and analysed using descriptive statistics in Microsoft Excel. - Free-text data were analysed qualitatively.	Qualitative (free text boxes) and quantitative (all other data)
Key informant, in-depth interviews	- Semi-structured interview guides, based on the programme theory, were used with patient- and carer-participants, RSWs, and HCPs who consented to participate. - Two trained qualitative Research Fellows (SR & AM) conducted telephone interviews with participants and HCPs after they had completed their intervention programme. - Interviews were audio-recorded using an encrypted recorder, anonymised, and transcribed for analysis. - All interviews were conducted exclusively by the two trained qualitative Research Fellows (SR and AM); no site staff were involved in data collection to ensure consistency and minimise potential bias.	Qualitative data
Focus groups	- Consenting HCPs participated in online focus groups using a Participatory Action Research approach [22]. - Sessions focused on implementation and how practitioners adapted and delivered the intervention collaboratively. - Session summaries were captured through notetaking.	Qualitative data
Training & supervision logs	- HCPs recorded their training and supervision throughout the trial. - Logs documented session duration, dates, and trainee/mentor initials.	Quantitative data
Audio recordings of sessions	- RSWs were instructed to audio-record selected intervention sessions, sampled for maximum variability. - Qualitative researchers assessed these remote, non-participant observations using a pre-developed protocol to measure process fidelity. - The protocol employed a rating scale to assess the presence, absence, or frequency of actions.	Quantitative data

### Data analysis

Quantitative data from CRFs, training, and supervision logs were analysed using descriptive statistics in Microsoft Excel and IBM SPSS Statistics v29. Non-participant observations via audio recordings were not completed due to lack of HCP consent and recording challenges.

All qualitative interviews were audio-recorded with participant consent, anonymised, and transcribed verbatim by an approved transcription company under a data-sharing agreement with the University of Exeter. Focus group sessions were documented in detail by a Research Fellow (SR) using structured note-taking templates. Transcripts and notes were uploaded to NVivo v14 for data management and analysis. NVivo was selected to support systematic organisation of data, enable structured application of the predefined coding framework, and facilitate comparison across data sources and fidelity domains.

A deductive framework analysis approach was used, guided by the modified Conceptual Model for Implementation Fidelity [7, 17, 32]. The framework informed the development of an a priori coding structure, which was iteratively refined to capture emerging nuances within each fidelity domain. Two researchers (SR and AM) independently coded a subset of transcripts to ensure consistency and credibility, resolving discrepancies through discussion and consensus. Regular analytic meetings were held with the wider team to refine the coding framework and interpretation. Coded data were summarised and charted within NVivo to identify recurrent patterns, refine domain-level themes, and develop cross-cutting insights related to fidelity and acceptability.

Analytic rigour was enhanced through maintenance of an audit trail documenting coding decisions, reflexive notes, and versioned codebooks. Input from the study’s PPIE group was incorporated during interpretation to ensure that participant perspectives were accurately represented.

The mixed-methods dataset was triangulated to consolidate findings across qualitative and quantitative sources, exploring different aspects of fidelity and acceptability. The categories of the modified implementation fidelity framework defined the triangulation table, and summaries from each dataset were compared to identify convergence, complementarity, and divergence in findings. A joint display table was developed to visually integrate qualitative and quantitative findings across each fidelity domain, enabling systematic comparison and supporting transparent interpretation of mixed-methods results.

### Implementation fidelity categories

We followed the stepwise approach to fidelity assessment within process evaluations as recommended by Carroll et al. [7] and Hasson et al. [17]:

1. A description of the intervention, its purpose, core inputs, expected mechanisms and desired outcomes were summarised in a logic model (see Table 1).
2. A detailed description of the intervention components, its delivery, dosage and implementation processes were detailed in an intervention manual.
3. General process questions were developed for each category and subcategory of the process evaluation based on the modified framework [7, 17], followed by a description of the data source(s) used to measure this category. This is presented in Table 2.

**Table 2:** Process evaluation categories, general process questions and corresponding data sources

Process evaluation category/subcategory	General process question	Data source(s)
Content	- Were each of the intervention components implemented as planned?	- CRF data - Participant interviews - Focus group feedback - Audio recordings of sessions
Dose, frequency, duration	- Were the intervention components implemented as often and for as long as planned? - Was the dosage received by participants appropriate and in line with the intended dosage?	- CRF data - Participant interviews - Focus groups
Coverage (reach)	- What proportion of target group participated in the intervention?	- CRF data - Participant interviews
Participant responsiveness	- How engaged were participants with the intervention services? - How satisfied were the participants with the intervention services? - How did the participants perceive the outcomes and relevance of the intervention? - What was the perspective of intervention facilitator on participant engagement, perception of value or recovery and satisfaction?	- Participant interviews - Focus group feedback
Recruitment & retention	- What constituted barriers to maintaining involvement of participants?	- CRF data - Participant interviews
Context	- What factors at political, economic, organizational, and work group levels affected the implementation?	- Participant interviews
Facilitation strategies	- What strategies were used to support implementation? - How were these strategies perceived by staff involved in the project?	- Participant interviews - Focus group feedback - Training and supervision logs
Intervention complexity	- How detailed or specific was the description of the intervention in the manual and/or training programme? - What were the perceptions of the facilitators regarding the intervention’s complexity?	- Participant interviews - Focus group feedback
Quality of delivery	- How appropriate was the delivery of the intervention by the facilitators in line with the logic model and programme theory?	- Audio recordings of sessions - Participant interviews

### Ethical approval

The feasibility study and embedded process evaluation was approved by the London – South East Research Ethics Committee (IRAS ID: 302675) and Scotland A Research Ethics Committee (IRAS ID 319555). All participants provided written informed consent prior to participating in this research. Names or other personally identifying information were removed from all data collected during transcription.

## Results

Nineteen participant–carer pairs consented to participate in the feasibility trial, which was lower than the target sample size specified in the study protocol for this feasibility phase [2] due to challenges with recruitment [1]. The demographic details of the patient and carer participants are presented in Table 3.

**Table 3:** Patient and carer participant demographics

Patient demographics (*N* = 19)		
Age (in years)	M = 85.0, SD = 6.5	
Sex	Male	12 (63%)
	Female	7 (37%)
Ethnicity	White British	19 (100%)
Number of hospital admission days	M = 33.9, SD = 18.9	
Discharge destination	Home	19 (100%)
Dementia status	Previously diagnosed with dementia	5 (26%)
	Not previously diagnosed with dementia	14 (74%)
Living arrangement	Lives alone	4 (21%)
	Lives with spouse/partner	11 (58%)
	Lives with other family member	4 (21%)
Carer demographics (*N* = 19)		
Age (in years)	M = 66.6, SD = 14.4	
Sex	Male	4 (21%)
	Female	15 (79%)
Ethnicity	White British	19 (100%)

The number of participants by category who consented, were interviewed and attended a focus group are detailed in Table 4.

**Table 4:** Process evaluation participation

Participant category	Consented (*N*)	Interviewed (*N*)	Attended a focus group (*N*)
Older people with delirium	8	5	NA
Carers	11	9	NA
Therapists (OTs, PTs)	7	4	5
RSWs	4	4	2
TOTAL	**30**	**22**	**7**

*N* Number of people, *NA* Not applicable

Healthcare professionals were recruited across all study sites except Birmingham, with representation from both rehabilitation support workers and therapists at most sites (Table 5).

**Table 5:** Recruitment of HCP participants by site

Study site	Rehabilitation Support Workers (RSW)	Therapists	Total
Birmingham	0	0	0*
Devon	1	1	2
Edinburgh	1	1	2
London (CNWL)	1	1	2
Newcastle	0	1	1
Nottingham	1	3	4
**Total**	**4**	**8**	**12**

* The study did not proceed at the Birmingham site; therefore, no healthcare professionals were recruited

### Adherence

#### Content

The manual recommends conducting the initial assessment within 14 days of discharge, but on average, it occurred after 18 days. This delay may be attributable to staff workload and scheduling challenges commonly experienced in NHS settings. All initial assessment parts were completed 69% of the time, providing an essential baseline of participants’ skills, abilities, preferences, and goals, consistent with our approach to setting personalised goals. 93% of planned sessions proceeded without interruption, referring specifically to scheduling.

**Fig. 3:**
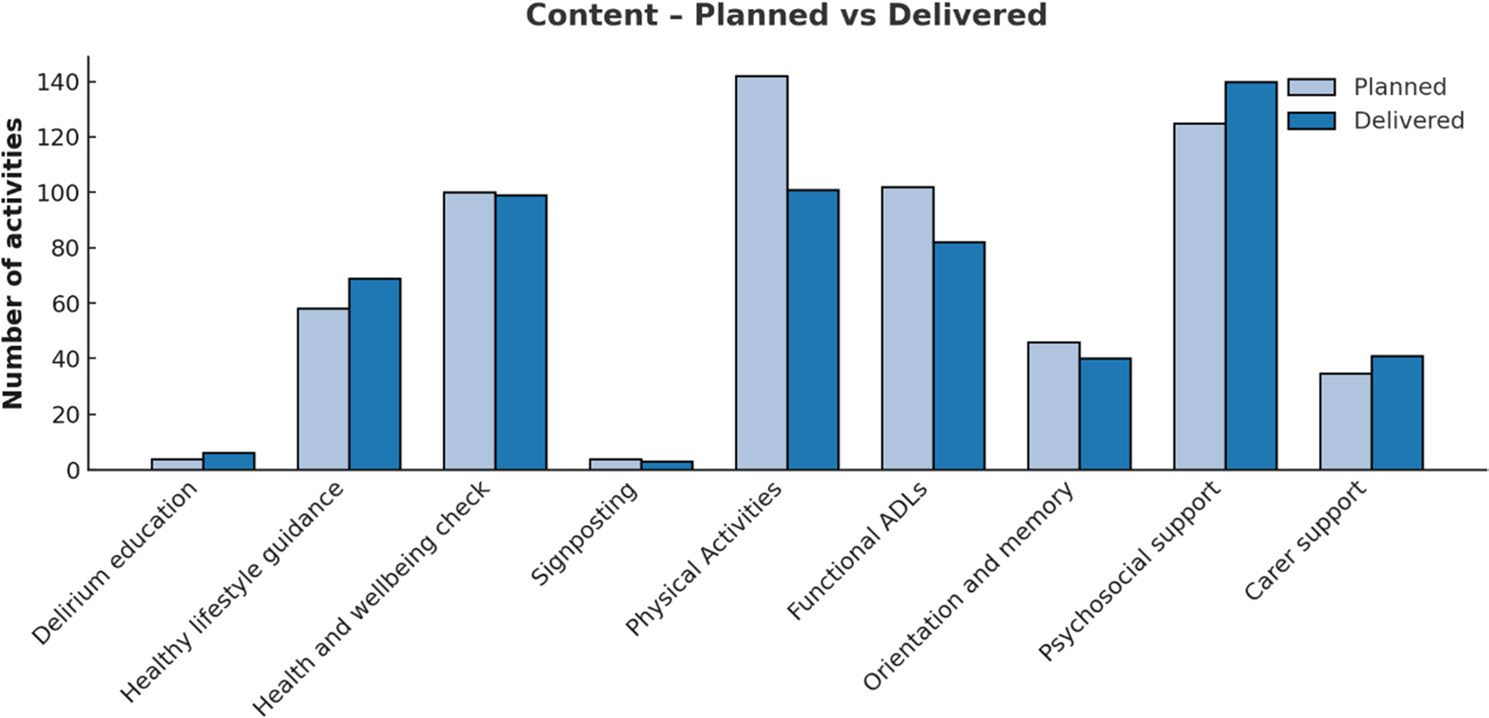
Activities planned versus activities delivered

As the intervention was tailored to individual needs, no two participants received an identical set of planned activities. Figure 3 illustrates the number of activities planned versus delivered across intervention components. Psychosocial support (+12.0%), healthy lifestyle guidance (+19.0%), and carer support (+17.0%) were delivered more often than planned, whereas physical activities (−29.0%), functional Activities of Daily Living (ADLs) (−20.0%), and orientation and memory activities (−13.0%) were delivered less frequently. Health and wellbeing checks were implemented largely as intended (−1.0%). These variations likely reflect differences in participant health, motivation, and session time constraints. Where ADLs or physical rehabilitation components were not delivered, this was usually the result of proactive adjustment to the participant’s health status, safety, or contextual circumstances, rather than the sessions being skipped entirely.

#### Dose, duration and frequency

All respondents (patients, carers, and professionals) generally found the dose and duration appropriate. The mean intervention length was 7.7 weeks (SD = 3.7), with participants receiving 8 sessions on average (SD = 2.9). Sessions lasted a mean of 58 minutes (SD = 21), and 10 participants (52.6%) completed the minimum required 6 sessions.

Most patient-participants valued ongoing contact with HCPs.

“It was good. It was longer than I'd expected, but I was grateful that it had been.” (Participant PC0006)

Professionals felt extended rehabilitation was necessary for meeting their goals and recovery.

“Up to ten sessions is brilliant, because community therapists generally offer just a couple, two or three max... and with delirium, having a longer period to recover is essential. All our participants needed that... and they all benefited from it. It then came to a natural pause when their next of kin and we felt they’d achieved what they wanted.” (HCP 4007)

Some professionals considered the duration appropriate due to supplementary community therapy, integrating well with existing care pathways. They noted session length varied based on individual needs and engagement. Some goals had to be adjusted as one-hour sessions were insufficient.

#### Coverage

The study aimed to recruit individuals over 65 with delirium. The mean participant age at discharge was 84.3 years; 63% were male. Although recruitment occurred across six NHS Trusts serving demographically diverse populations, eligible participants during the recruitment period were predominantly White British. Recruitment procedures relied on identification through hospital delirium teams and the presence of an informal carer able to participate, which may have unintentionally limited inclusion of participants from minority ethnic backgrounds.

*“The city where we live is incredibly diverse city in terms of race*,* ethnicity*,* religion. I was slightly surprised there wasn’t more participants from a wider variety there. Whether that was just representative of what was turning up at the acute hospital during the research project*,* or whether there [were] other barriers there to engaging with the research*,* I’m not sure.” (HCP 6002)*.

Only 26% had a prior dementia diagnosis, with one new diagnosis during the study. Some professionals felt participants’ delirium had mostly resolved by the start of the intervention, while others had complex needs beyond the scope of RecoverED, such as mental health issues or awaiting surgery impacting mobility, or probable dementia, suggesting the intervention was insufficient for their recovery.

*“The participant who ended up going into a care home*,* I remember going in there and I [thought] ‘wow*,* this is going to be really*,* really challenging’. Because*,* I think*,* the main challenge was*,* I couldn’t engage her with conversation.” (HCP 6002)*.

### Moderating factors

#### Participant responsiveness

Participant responsiveness was explored through satisfaction, engagement, and perceived value. Most participants and carers reported a positive experience, appreciating the one-on-one approach and home-based delivery.

Professionals received high praise for their encouragement and motivation in delivering the intervention.

*“[The professionals] made a point of saying that they were quite astounded at the progress I’d made in the period of care*,* because I had gone from being a fairly poorly looking person on being discharged from hospital; within sort of some weeks*,* I was actually just performing in the general sense far better. They liked to tell me how and where I was getting better. And they were doing it very positively.”* *(Participant PC02)*.

Relationship continuity, a key aspect of RecoverED’s delivery, reinforced the importance of trust and rapport between care recipients and HCPs. Carers also felt well-supported and valued having someone to discuss their own experiences with.

Professionals observed good engagement, with participants receptive to guidance, actively involved in rehabilitation activities, and carers participating, particularly in physical and mobility exercises. While some participants remained focused and willing to take risks, others were less engaged due to low motivation, reluctance to take risks, or frustration with slow progress. Some were especially hesitant to engage in psychosocial activities, health and wellbeing checks, and healthy lifestyle guidance.

Regarding the perception of value, many saw RecoverED as filling a gap in community care.

*“If we hadn’t agreed to take part in this*,* I just don’t think we would have had any support of any sort. We’ve had medical care based in hospital*,* we’ve had physiotherapy*,* but there [would have] been nothing on the mental side at all.”* *(Carer PC10)*.

Many general benefits were noted; patients regained skills and interests, improved cognitively and functionally, and achieved goals.

*“By the time another week came*,* I’d passed the goals*,* and I was on to another goal. I had my own goals for timing myself walking around the block. I went shopping at the supermarket on my own with the kids and they were really pleased with it. From week to week*,* I could see improvement. Physically and mentally*,* yes.” (Participant PC08)*.

Professionals saw many participants make good progress on each domain but also in terms of attaining their pre-set goals.

*“We managed to get his mobility back up to where he would like it. So yeah*,* he did get on with it well. And cognitively*,* his daughter would always say to me*,* ‘I’ve got my dad back*,* I’ve got my dad back*,* we’re back to where we were*,* I’ve got him back*,* he’s wonderful.‘” (HCP 2001)*.

Some participants required additional support beyond RecoverED due to significant impairments or complex care needs, with four participants moving to care homes or palliative care.

#### Recruitment

Recruitment posed significant challenges [1]. Only 9% (*N* = 36) of those screened (*N* = 419) were eligible, of whom 53% (*N* = 19) consented. This affects coverage, which examines the proportion of eligible participants who took part in the study.

Some participants experienced delays between recruitment and intervention, meaning their delirium had mostly resolved. This raises the question of whether the generally positive views on the intervention dose were influenced by the mildness of symptoms in many participants. Others had complex needs beyond RecoverED’s scope.

Of 19 participant-pairs, 10 (53%) withdrew due to care home placement (*N* = 4), unrelated death (*N* = 2), deterioration (*N* = 2), lack of benefit (*N* = 1), or prolonged intermediate care (*N* = 1). This reflects the severity of many participants’ health conditions, complicating the interaction between adherence to the intervention and recruitment.

#### Context

RecoverED was delivered by multidisciplinary teams (MDTs) within the NHS, providing insights into the considerations necessary for implementing it in real-world settings. HCPs had to balance training, planning, and delivering the intervention alongside their regular NHS workloads, often putting in additional hours. Staffing shortages at most sites made organising the teams challenging, and many struggled to assign the required professionals. Without dedicated teams, some professionals felt the intervention would not be feasible, as it was time-consuming and difficult to coordinate.

*“We haven’t got our physiotherapist as part of the RecoverED program*,* just because we can’t really afford to have that much time off for them. We would need a dedicated team; we would need a separate pathway to be able to [deliver RecoverED]. (HCP 4001)*

Professionals stressed the importance of geographical accessibility to assigned participants, given the complexities of delivering weekly or bi-weekly hour-long sessions. They also emphasised that RecoverED teams should comprise HCPs who are familiar with each other and share similar working styles, as the intervention depends on consistent MDT collaboration, which can be difficult with unfamiliar colleagues. Additionally, they noted that teams should be geographically co-located to facilitate seamless planning, debriefing, and supervision of sessions.

*“If I had been working within a pre-existing team… you’d most likely see the therapists that were involved ask*,* ‘how was it? Let’s have a quick debrief.’ Whereas I never had that*,* and I felt like I was working in isolation.’* *(HCP 3001)*.

RSWs in the study were typically Band 3 or Band 4 skilled, non-registered staff. In the NHS, Band 3 refers to lower-level support roles, while Band 4 involves more advanced skills and responsibilities, though still non-registered. RSWs felt they required training, guidance, and supervision from therapists or study teams to confidently tailor and deliver the intervention.

#### Facilitation strategies

##### Training & manual

Professionals praised the comprehensiveness of the RecoverED training programme and intervention manual, noting its value beyond the programme itself.

*“I would say it boosted my confidence; it made me more aware. I didn’t really know much about delirium. But [the training] has given me more information*,* so*,* from doing your delirium training and learning more about it I [can] see straightaway*,* ‘this person clearly has got an infection*,* and it is potential delirium because of this’.”* *(HCP 6001)*.

The pre-recorded, self-paced training package was accessed an average of 4 times (SD = 1.3), indicating it was completed over several sessions. This aligns with professionals’ feedback on the challenge of fitting training around their regular workload. While most found the manual thorough, some felt it was lengthy and repetitive.

*“[The manual] was a bit repetitive and very long*,* and so most people did it from home because it [took] six or seven hours to get through all the modules*,* and in a busy NHS environment*,* it’s hard to get your mandatory training completed*,* let alone additional training.”* *(HCP 4007)*.

There were requests for more hands-on, practical training, face-to-face sessions, and better organisation of materials to suit different learning styles and access needs.

*“It would have been good to see some small video clips of it in action.”* *(HCP 3002)*.

*“Having one-to-one training with a person talking it through… because I’ve got dyslexia. So*,* for me to sit there and just read it through wasn’t very helpful.”* *(HCP 6001)*.

##### Peer support and supervision

Training and supervision logs did not clearly outline how internal team supervision was structured to ensure seamless planning and delivery across sites. However, interviews highlighted that team member compatibility and familiarity were crucial for effective intervention delivery.

Peer support sessions were held online throughout the implementation period to provide opportunities for reflection and shared learning across sites. Invitations were circulated to all staff involved in intervention delivery, but attendance was not mandatory. Participation varied between sites depending on staff capacity and workload.

The study team held seven peer support sessions at an average interval of 37 days (SD = 19.4), with a mean attendance of six participants (SD = 2.1); all sites participated in at least one session. Feedback was generally positive, although a more structured format with predetermined discussion topics was suggested to enhance their value. Ad hoc support from the study clinical team was also widely accessed and highly valued.

*“It was good to have access to the clinicians on the study team*,* to sort of run things by. That… was probably the greatest resource.”* *(HCP 3002)*.

#### Intervention complexity

RecoverED is a novel, person-centred, multicomponent intervention addressing diverse rehabilitation needs. Professionals emphasised the MDT approach, noting how interactions between components were shaped by participants’ abilities and functional recovery goals.

*“Everything was very function based*,* and goal related. So*,* the physical*,* cognitive and psychosocial*,* they all impacted on each other… working on physical limitations and then cognitive limitations and kind of improving their mood because they were making [meals] for their wife.”* *(HCP 4007)*.

Some professionals were unsure about the permissible extent of tailoring and adaptation and how to plan and deliver multiple components. They sought clearer guidance and noted that there was a learning curve with the manual and training.

#### Quality of delivery

##### Person-centred care and tailoring

Interviews indicated that the intervention aligned with person-centred care principles. Professionals were seen as prioritising participants’ best interests to optimise progress and recovery.

*“They discouraged [the participant] from feeling like you’re a poorly person that can’t do anything. And then encouraged you to be looking towards making progress and gave you goals as well.”* *(Carer PC08)*.

Patients and carers valued how professionals incorporated participants’ backgrounds, occupations, and preferences into rehabilitation activities. Crucially, goals were participant-led, allowing individuals to make their own care decisions.

*“Carer: The walking thing was a very big important thing for Dad. And she said*,* ‘Right*,* what goal shall we set?’ And we said*,* ‘Oh*,* he likes to sit down on the bench in the garden.*

*Participant*: *And we walked up to that bench*,* yeah.*

*Carer*: *She wrote those goals down*,* and every week we worked towards them. And he has achieved them. So*,* it did work… and you need somebody who says*,* ‘Oh*,* yeah*,* you can do that*,* we’ll do that!‘”* *(Participant & Carer PC04)*.

Professionals adapted goals to keep them and achievable. Midway reviews were used to assess progress and refine goals, which evolved over time. Session length and duration were also adjusted based on participants’ goals and the complexity of their needs.

##### Carer involvement

Carers were present during the initial assessment in 92% of cases and involved in subsequent sessions 70% of the time. Their level of involvement varied, with greater participation in physical rehabilitation. Those with a medical background were particularly motivated to engage. Open-plan environments and working from home also facilitated their involvement. Conversely, some carers chose not to participate, either because they felt it was unnecessary or preferred to let the intervention run its course independently.

*“I just pushed off and let [participant] get on with it… at the beginning… I hovered an awful lot. But it was their level of professionalism… I didn’t feel I needed to be included in things that were for him and his legs and his strength and so on.”* (*Carer PC08)*.

*“I tended to leave them. I didn’t interfere in any way. I felt it was more important to allow that to take its own course*,* and to allow my husband to have interaction with another person apart from me.”* *(Carer PC10)*.

##### Relationship continuity

Participants valued the continuity of care with HCPs, contrasting it with previous challenges of having multiple carers. Many felt they had built a strong rapport, even describing friendships, and were saddened when the intervention ended. Some expressed a desire to stay in touch.

*“The social conversations that were not necessarily related to [delirium] were quite important*,* because [the professionals] became our friends. We felt the need to hug them… so there we go.”* *(Carer PC08)*.

This was also reflected in professionals’ views of their own experience.

*“With the first pair*,* and it was quite sad saying goodbye at the end. With the second pair*,* based on some of the feedback I’ve had just from [the carer]*,* she’s been very grateful for the input.”* *(HCP 3001)*.

Building trust and rapport took time but was essential for quality care. It encouraged participants to attempt challenging rehabilitation activities and enabled professionals to tailor the intervention based on participants’ preferences. Some professionals saw this as key to successful outcomes.

*“I was quite amazed at how readily*,* willing people were to accept you in*,* and trust you*,* and get going with you… I think if you put somebody in there who was just going in with a very dry clinical approach*,* I don’t think that would have a good outcome*,* whereas the [interpersonal relationship] was fundamental… that’s been the defining feature in a way.* *(HCP 3001)*

##### Integration to daily activities

The intervention was goal-centred, with patients setting goals based on their daily life priorities. This patient-centric, goal-based approach aligned with the OT-based rehabilitation model familiar to many professionals. Participants appreciated that the rehabilitation was structured around their functional recovery goals, supporting their independence at home.

*“I can do exactly what I want to; I can make my own bed; I can look after myself and cook a good dinner myself. In fact*,* on Sunday*,* I cooked a nice roast dinner. And it was absolutely wonderful. That’s how I like to live.”* *(Participant PC04)*.

##### Integration with usual services

Participants felt that receiving RecoverED alongside community therapy or social care immediately after discharge supported their recovery. However, coordinating multiple support streams required careful planning. MDT teams were seen as essential for delivering RecoverED.

*“RecoverED may be better done with a community team*,* and getting on board the whole MDT*,* so to have a GP involved or a virtual ward involved might be useful. So*,* maybe having a RecoverED team that is allocated a GP who could discuss cases*,* an OT*,* a physio*,* a nurse and a rehab assistant would be good to a tailored team who can support the RecoverED interventions.”* *(HCP 4007)*.

Professionals frequently signposted participants to other services, such as GPs, rapid response teams, old age psychiatry, and social care, when needs exceeded the intervention’s scope. Awareness of existing services that can complement RecoverED is crucial for effective implementation.

*“I think a lot of the issues that were affecting [the participant] were long-standing and predated her admission and her experience with delirium*,* I definitely identified serious issues with mood and anxiety which then led me to refer to our GP for urgent review*,* which is where we’re at now*,* and there’s old-age psychiatry input now.”* *(HCP 3001)*.

## Discussion

This study presents the embedded process evaluation of the RecoverED intervention conducted alongside a feasibility study. Overall, the intervention was delivered with good fidelity, and both participants (older people with delirium and their carers) and professionals (OTs, PTs, RSWs) found it acceptable and valuable. The fidelity and acceptability of the intervention were influenced by its multicomponent and flexible design, which necessitated tailoring to individual needs.

NICE guidelines recommend that multicomponent interventions to prevent delirium must be tailored to the person’s needs [29]. Capturing data on tailoring and adaptations of multi-component interventions can be a complex, multi-stage process [20]. As predefined, the intervention was highly tailored, with professionals planning activities based on each participant’s goals. This demonstrates fidelity to ‘function’, which is extent to which the intervention was tailored and adapted in a person-centred manner [19].

Some components, such as psychosocial support, was delivered more often than planned, highlighting its importance in delirium recovery. Research links delirium to PTSD-like symptoms, particularly after prolonged hospitalisation or ICU stays [15, 16, 39], and to more severe depression in people with hip fractures [28]. This study found some HCPs were less familiar with providing psychosocial support, reflected in both interviews and lower rates of planned sessions. The EnCOP workforce competency framework stresses the need to address older people’s psychological and social needs, particularly in delirium. Effective psychosocial care requires a broad skill set, reinforcing the need for training and supervision to help HCPs manage psychosocial risks after discharge [38].

Overall adherence to dose was moderate, with participants completing, on average, around eight 1-hour sessions. However, HCPs noted that many participants had nearly or fully recovered by the time they started. As a result, those who completed the intervention with good adherence to the intended dosage may not fully represent the complex rehabilitation needs RecoverED aims to address. Participants with more complex health issues withdrew for reasons unrelated to the intervention or trial, including deteriorating health, care home placement, or death, limiting insight into adherence in this group. This suggests the intervention may be better suited to those with less complex needs while highlighting the need to refine our understanding of the population most likely to benefit from RecoverED.

The intervention was well-accepted by those who completed it, with high satisfaction reported by both participants and professionals. However, the views of those who withdrew were not captured as part of the process evaluation. Based on their reasons for withdrawal, which were largely unrelated to the intervention, it is clear that the intervention is generally acceptable but may not be appropriate for individuals with more severe impairments.

Quality of delivery, while generally aligned with the intervention’s theory, was complicated by practical challenges, such as the lack of non-participant observations due to consent issues and practical constraints. Previous studies have documented similar challenges with observations through audio-recording sessions [40, 41], although other studies have successfully implemented this approach [12, 34].

Evaluation of intervention complexity was closely linked to facilitation and support processes. Healthcare professionals reported generally positive experiences of the training programme and manual but consistently emphasised the importance of ongoing peer and ad hoc support in delivering this complex, personalised intervention. Insights derived from interviews and focus groups highlighted uncertainties around intervention planning and delivery that were often addressed through informal peer discussion and ad hoc support, rather than through manualised guidance alone. This suggests that while the training manual provided a necessary foundation, manualised training by itself may be insufficient for complex, tailored interventions, and that planned mechanisms for ongoing peer and ad hoc support should be embedded as a core component of future implementation models.

Feedback on peer review sessions was generally positive, but the effectiveness of study-specific supervision was unclear based on available data. The need for dedicated, co-located teams familiar with each other was emphasised, as this would facilitate more efficient and collaborative MDT working. Additionally, assigning participants closer to their HCPs’ workplace could improve the intervention’s delivery efficiency. Although workload pressures and staffing shortages were reported across participating sites, these factors did not appear to compromise the fidelity of intervention delivery. These findings should be interpreted cautiously, given the smaller-than-anticipated recruitment and the resulting limits on representativeness of routine delivery conditions.

A methodological strength of this study is the use of a widely utilised framework to evaluate implementation fidelity and acceptability [7, 17]. The framework is empirically and practically useful in understanding how adherence and moderating factors interact and in identifying areas for improvement or modification. The use of the Conceptual Model for Implementation Fidelity provided a robust structure for assessing adherence, moderating factors, and quality of delivery. Our findings support the model’s utility in identifying contextual influences and strategies that help maintain fidelity in complex, person-centred interventions. In line with MRC guidance on developing and evaluating complex interventions, this process evaluation demonstrates how theoretical fidelity assessment can inform future implementation strategies, ensuring that adaptations remain evidence-based while preserving core intervention functions.

### Limitations

Long-term follow-up was not conducted within this process evaluation, as it was embedded within a feasibility study. Previous research has shown that fidelity and acceptability may change over time [12, 26, 27], although many of these studies were conducted alongside RCTs. Future evaluations of the RecoverED intervention within RCTs should therefore incorporate longitudinal data collection methods.

High dropout rates and the resulting sample bias limit the extent to which fidelity and acceptability can be fully assessed, as participants who remained in the study may differ meaningfully from those who withdrew.

As a novel intervention delivered within a feasibility study, implementation support was provided through ad hoc and peer support sessions. While this support may have influenced implementation fidelity, potentially limiting generalisability to wider practice, it was necessary at this developmental stage to ensure consistency and learning across sites.

The study did not recruit participants from minority ethnic, racial, or linguistic groups, limiting the generalisability of the findings, particularly in relation to acceptability for these groups. This underrepresentation likely reflects both the eligibility criteria and recruitment processes used in this feasibility phase, which may have unintentionally constrained inclusion. It also highlights that issues of equity, access, and cultural relevance should be considered when interpreting the study’s implications for wider implementation. Future trial phases will address this through expanded engagement strategies and collaboration with community and voluntary sector organisations to improve inclusion of underrepresented groups.

## Conclusion

The RecoverED intervention was well-received, with participants’ reports of perceived benefits, including improvements in independence and functional recovery, supporting its acceptability. As a complex, person-centred intervention, RecoverED requires careful tailoring and planning to align with participant-led goals. Delivery of the psychosocial component may require additional capacity building within delivery teams, though overall quality of delivery was high and consistent with the intervention’s theoretical foundations.

Our findings suggest the need to refine eligibility criteria and recruitment strategies to better identify the target population, particularly those with ongoing or complex recovery needs. These insights will inform optimisation of dosage, coverage, and implementation strategies in a future RCT nd guide planning for wider roll-out within community rehabilitation settings. Future efforts to refine and implement the intervention should focus on supporting people with deteriorating health or those requiring care home placement.

## Data Availability

Data are available upon reasonable request.
